# Protection of SPF Chickens by H9N2 Y439 and G1 Lineage Vaccine against Homologous and Heterologous Viruses

**DOI:** 10.3390/vaccines11030538

**Published:** 2023-02-24

**Authors:** Hyun-Kyu Cho, Yong-Myung Kang, Mingeun Sagong, Juhun Kim, Hyunjun Kim, Sungjun An, Youn-Jeong Lee, Hyun-Mi Kang

**Affiliations:** 1 Avian Influenza Research & Diagnostic Division, Animal and Plant Quarantine Agency, 177 Hyeoksin 8-ro, Gimcheon-si 39660, Republic of Korea; 2 Bioapp Institute, 394 Jigok-ro, Pohang-si 37668, Republic of Korea

**Keywords:** vaccine, avian influenza, H9N2, protection, multiple passage

## Abstract

Prior to the identification of low pathogenic avian influenza H9N2 viruses belonging to the Y280 lineage in 2020, Y439 lineage viruses had been circulating in the Republic of Korea since 1996. Here, we developed a whole inactivated vaccine (vac564) by multiple passage of Y439 lineage viruses and then evaluated immunogenicity and protective efficacy in specific-pathogen-free chickens. We found that LBM564 could be produced at high yield in eggs (10^8.4^EID_50_/0.1 mL; 1024 hemagglutinin units) and was immunogenic (8.0 ± 1.2 log_2_) in chickens. The vaccine showed 100% inhibition of virus in the cecal tonsil with no viral shedding detected in either oropharyngeal or cloacal swabs after challenge with homologous virus. However, it did not induce effective protection against challenge with heterologous virus. An imported commercial G1 lineage vaccine inhibited viral replication against Y280 and Y439 lineage viruses in major tissues, although viral shedding in oropharyngeal and cloacal swabs was observed up until 5 dpi after exposure to both challenge viruses. These results suggest that a single vaccination with vac564 could elicit immune responses, showing it to be capable of protecting chickens against the Y439 lineage virus. Thus, our results suggest the need to prepare suitable vaccines for use against newly emerging and re-emerging H9N2 viruses.

## 1. Introduction

H9N2 low pathogenic avian influenza (LPAI) viruses have been circulating and have become prevalent in poultry worldwide [1,2,3,4]. Infections result in large economic losses; therefore, vaccination against H9N2 has been adopted by countries in which H9N2 is endemic in poultry (e.g., China, Vietnam, the Middle East, and Korea) [2,5,6,7]. H9N2 viruses are divided into three lineages: G1, Y280, and Y439; the G1 lineage viruses are the most widespread throughout India, Pakistan, the Middle East, and North Africa [2,8,9,10]. The Y280 lineage viruses, found primarily in China, are extremely diverse [11,12]. In particular, Y280 and G1 lineage viruses have acquired specificity for receptors on human cells, making them a public health concern [13,14,15,16,17]. Y439 lineage viruses have been isolated mainly in Korea, but occur sporadically in poultry in Eurasia, including China and Vietnam [3,10,18].

In Korea, Y439 lineage H9N2 viruses have been isolated since 1996; they caused apparent clinical signs, especially a drop in egg production from 80% to less than 5%, with 20–40% mortality in 1996 [4,19,20,21]. Between 1996 and 1999, the Korean government engaged in “stamping-out” and compensation policies and then began using an inactivated vaccine in 2007 [4]. Initially, vaccination reduced the incidence of infection by Y439 lineage H9N2 viruses; indeed, viruses were isolated mainly to unvaccinated live bird markets or from Korean native chickens [4,22]. Although the virus has not been isolated since June 2018, a Y280 lineage H9N2 virus that is distinct from the previously endemic virus has been circulating in Korea since June 2020 [23,24]. In particular, Y439 lineage H9N2 virus is known to replicate preferentially in the gastrointestinal tract, whereas Y280 lineage H9N2 viruses replicate mostly in the respiratory tract and spread rapidly under laboratory-controlled conditions [25]. In one study, they showed mortality with 15 birds dying in 1 day and reducing egg production from 10 to 27% in the field [24]. Hence, a recombinant vaccine candidate against Y280 lineage virus (rgHS314) was developed in Korea [26,27].

Current vaccine research aims to reduce the cost and difficulty of production of current inactivated vaccines, while also increasing efficacy and providing broad protection against multiple H9N2 subtypes [2]. Recent studies report development of H9N2 recombinant vaccines generated by reverse genetics; these vaccines contain the internal genes of the high yield PR8 strain (A/Puerto Rico/8/34 (H1N1) [26,28]. Additionally, H9N2 vectored vaccines based on Newcastle disease virus, fowl pox virus, and turkey herpesvirus have been developed [29,30,31]. Another study reported generation of an H9 virus-like-particle vaccine using a non-egg-based platform [32]. Traditionally, vaccines against avian influenza viruses are derived from influenza isolates grown by multiple passage in embryonated chicken eggs; this results in high yield strains, which are then inactivated and delivered with mineral oil adjuvant [2,33,34,35]. 

In the current study, to prepare for the sudden introduction of H9N2 viruses, we developed a conventional whole inactivated vaccine by multiple passage of a recently isolated virus belonging to the Y439 lineage (A/Korean native chicken/Korea/LBM564/2017 (H9N2)). We then examined its immunogenicity and protective efficacy against challenge by Y439 and Y280 lineage H9N2 viruses. Moreover, we evaluated a commercial whole inactivated G1 lineage-based vaccine imported from the United Arab Emirates to assess its ability to protect chickens against challenge with the Y439 and Y280 lineage viruses. 

## 2. Materials and Methods

### 2.1. Viruses and Vaccines 

This study used two lineages of the H9N2 virus as challenge strains: A/Korean native chicken/Korea/LBM564/2017 (H9N2), which includes the Y439 lineage (hereafter LBM564), and A/Korean native chicken/Korea/LBM314/2020 (H9N2), which includes the Y280 lineage (hereafter LBM314). Both viruses were isolated by the Animal and Plant Quarantine Agency (APQA) of the Republic of Korea through a nationwide surveillance program. One vaccine was generated by multiple passage of the LBM564 strain (known hereafter as vac564) in embryonated chicken eggs. Another vaccine, a commercial H9N2 vaccine (CEVA, Libourne, France), was imported from the United Arab Emirates. 

### 2.2. Statistical Analysis

To examine vaccine efficacy, data from the vaccination and sham groups were subjected to statistical analysis performed using Prism 5 software (GraphPad, La Jolla, CA, USA). Data were compared using Fisher’s exact test and Student’s *t*-test, and *p*-values of 0.05 and 0.01 were considered significant.

### 2.3. Study 1: Protection of vac564 Vaccine against Challenge by Homologous and Heterologous Viruses in SPF Chickens

#### 2.3.1. Vaccine Candidate Development by Multiple Passage

The vaccine was developed by serial passage of LBM564 in 9–11-day-old embryonated specific-pathogen-free (SPF) eggs for 2–3 days at 37 °C; the virus was passaged 40 times to obtain high growth and productivity. The 50% egg infectious dose (EID_50_) was periodically measured as described previously [36]. To determine the optimum growth conditions for the vaccine, time-specific productivity of the virus (thirtieth passage, CE30) was investigated at different doses (10^2.0–5.0^EID_50_/0.1 mL) until 72 h. Specifically, inoculated eggs were killed at 12 h intervals, and virus titers were measured from 12 to 72 h in a hemagglutination activity (HA) assay. 

To clarify the minimum immunizing dose, immunization efficacy was examined at different vaccine doses (CE30; 10^7.5–8.0^EID_50_/0.1 mL). Briefly, each group of eight chickens were immunized with 10^7.5^ and 10^8.0^EID_50_/0.1 mL. Next, serum was obtained at 3 weeks post-vaccination (wpv) and tested in hemagglutination inhibition (HI) assays with 1% chicken red blood cells (RBCs). Finally, the virus was inactivated by exposure to 0.1% formalin (*v*/*v*) and stored at 20 °C for 18 h. Inactivation was confirmed in SPF eggs. 

#### 2.3.2. Vaccination and Challenge of SPF Chickens 

To evaluate the efficacy of the vaccine against challenge with homologous (LBM564) and heterologous virus (LBM314), SPF chickens (5-week-old) were divided into four groups (sixteen chickens per group): one immunization group and one non-immunization (sham) group for two challenge viruses. Vaccinated birds were injected intramuscularly (IM) with 0.5 mL of vac564 (10^8.0^EID_50_/0.1 mL) mixed with the Montanide ISA VG70 oil adjuvant (SEPPIC, Courbevoie, France). The sham groups were inoculated with PBS plus Montanide ISA VG70. At 3 wpv, all chickens were challenged intranasally (IN) with 100 µL of two H9N2 viruses (LBM564 or LBM314; each at 10^6.0^EID_50_/0.1 mL).

#### 2.3.3. Serology and Antibody Persistence

Blood samples were collected from all living chickens at 3 wpv and 14 dpi. HI assays were performed to measure antibody titers against 4 HA units of homologous antigen in the presence of 1% chicken RBCs. To examine the persistence of antibodies generated by vac564, ten chickens were immunized with a single dose of 10^8.0^EID_50_/0.1 mL with the Montanide ISA VG70. Serum was collected at 3, 8, 12, 20, and 24 weeks (6 months) post-vaccination, and antibody titers were measured by HI assays.

#### 2.3.4. Virus Shedding and Replication 

To examine virus shedding by infected chickens, eight chickens from each group were monitored daily until 14 dpi. Oropharyngeal (OP) and cloacal (CL) swab samples were collected from all groups at 1, 3, 5, 7, 10, and 14 dpi. To examine virus replication, eight chickens from each group were sacrificed at 5 dpi, and tissue samples (lung, trachea, cecal tonsil, spleen, kidney, and liver) were collected aseptically and stored at −80 °C until use.

Each sample was resuspended in 1 mL of maintenance medium containing antibiotic–antimycotic mixture (Invitrogen, Carlsbad, CA, USA). Samples were injected into 9–11-day-old embryonated commercial eggs, and virus growth was examined in standard HA assays. Viral titers were calculated using the Reed and Muench method [37]. The detection limit of the assay was 10^1.0^EID_50_/0.1 mL.

### 2.4. Study 2: Protection of Commercial G1 Lineage Vaccine against Challenge by Heterologous Viruses in SPF Chickens

#### 2.4.1. Vaccination and Challenge of SPF Chickens

To evaluate the protection of commercial G1 vaccine against challenge with heterologous viruses (LBM 564 and LBM314), SPF chickens (5-week-old) were divided into four groups as described above. Later, vaccines were administered IM in accordance with dosage guidelines (0.5 mL/bird). The sham groups were inoculated with PBS. At 3 wpv, all chickens were challenged IN with 100 µL of two heterologous viruses (LBM564 or LBM314; each at 10^6.0^EID_50_/0.1 mL).

#### 2.4.2. Serology and Antibody Assays

Serums were collected at 3 wpv and at 14 dpi. HI assays were performed to determined antibody titers, against 4 HA units of heterologous antigens (challenge virus as antigen; in the G1 vaccination, only heterologous viruses were used because no homologous virus was isolated in Korea) with 1% chicken RBCs. Hence, neutralizing antibody titers were additionally measured in serum neutralization (SN) assays; serum was diluted 1:10 and then serially diluted 2-fold. It incubated with 100 TCID_50_ (50% tissue culture infectious dose) of challenge virus for 1 h at 5% CO_2_/37 °C. After that, DF-1 cell (1.0 × 10^5^ cells/plate) was incubated with the serum mixture in 96-well plates for 72 h at 5% CO_2_/37 °C. Neutralizing antibody titers were defined as the highest dilution of serum that completely neutralized 100 TCID_50_ of virus.

#### 2.4.3. Virus Shedding and Replication

To examine virus shedding, OP and CL swab samples were collected from eight chickens from each group at 1, 3, 5, 7, 10, and 14 dpi. To examine virus replication, eight chickens from each group were sacrificed at 5 dpi, and tissue samples (lung, trachea, cecal tonsil, spleen, kidney, and liver) were obtained and stored at −80 °C until use. Samples were injected into 9–11-day-old embryonated eggs, and virus growth was examined in standard HA assays. Viral titers were calculated using the Reed and Muench method [37]. The detection limit was 10^1.0^EID_50_/0.1 mL.

## 3. Results 

### 3.1. Study 1: Protection of vac564 Vaccine against Challenge by Homologous and Heterologous Viruses in SPF Chickens

#### 3.1.1. Development and Characteristics of Vaccine Candidate

**Figure 1:**
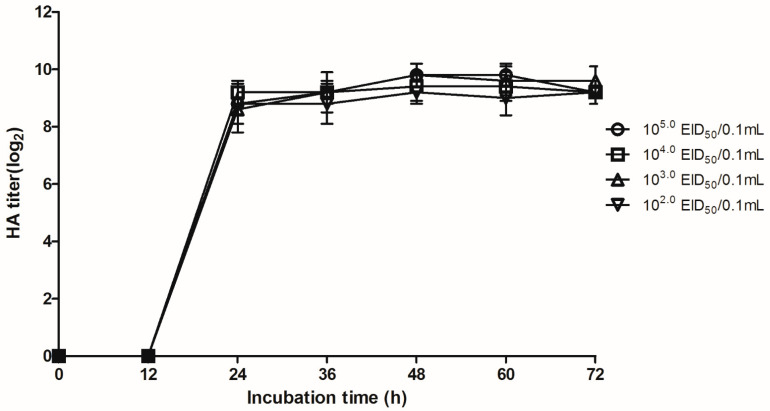
Growth of vac564 (10^2.0^–10^5.0^EID_50_/0.1 mL) in specific-pathogen-free (SPF) eggs over time. Error bars indicate standard deviation of the hemagglutination activity (HA) titers (*n* = 5) by inoculating doses.

The vaccine was developed by serial passage of virus (LBM564) in eggs. The virus propagated stably, reaching 1024 HA units and an EID_50_/0.1 mL of 10^8.4^ at passage 30 was then selected as the final product (vac564) (Table 1). In terms of time-specific productivity, vac564 propagated well for 36–72 h without killing the embryonated SPF eggs; the HA titer peaked at 9.8 ± 0.4 (log_2_) at 48 h (Figure 1). The minimum immunizing dose was 10^8.0^ EID_50_/0.1 mL, which yielded an HI titer of 8.0 ± 1.2 log_2_ in SPF chickens at 3 wpv (Table 2).

#### 3.1.2. Serological Responses and Antibody Persistence

**Figure 2:**
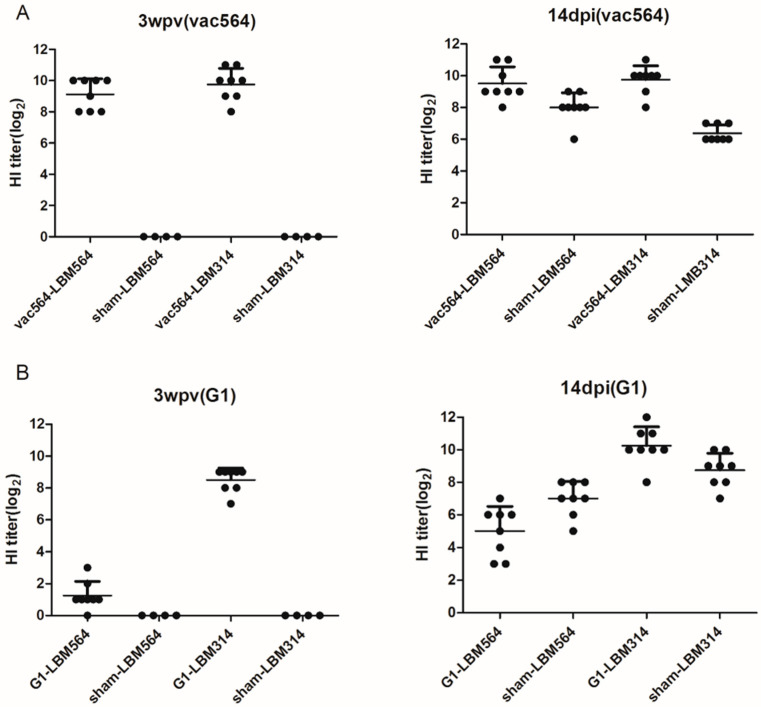

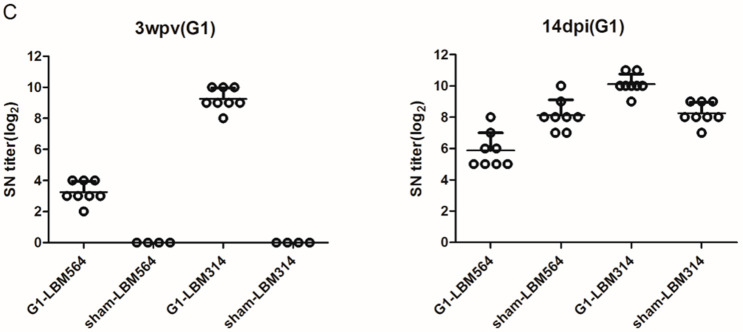
Antibody responses induced by vac564 and G1 lineage vaccines at 3 weeks post-vaccination (wpv) and at 14 days post-infection (dpi). Hemagglutination inhibition (HI) and serum neutralization (SN) assays were performed. (**A**): vac564 vaccination-challenged group. Black circles indicate the HI titers. (**B**): G1 vaccination-challenged group. Black circles indicate the HI titers. (**C**): G1 vaccination-challenged group. White circles indicate the SN titers. For serum analyses, G1 used the challenge strain as the antigen.

**Figure 3:**
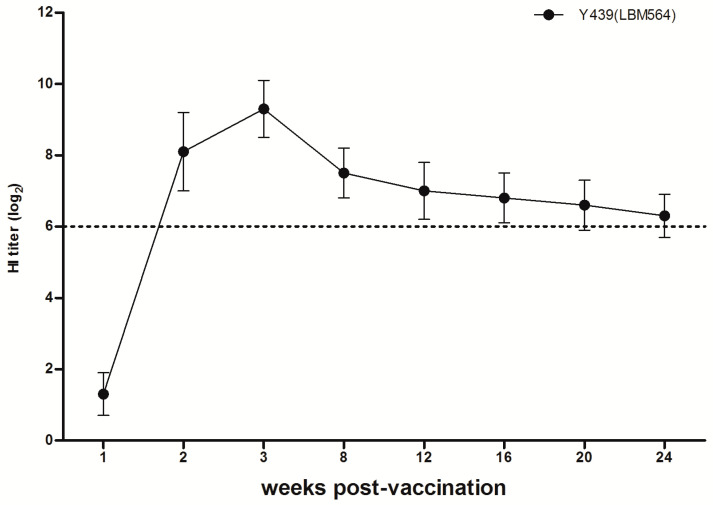
Antibody persistence after vaccination with vac564. Hemagglutination inhibition (HI) titers were examined up to 24 weeks post-vaccination (wpv). vac564 induced HI titers of >6 log_2_ at 2–24 wpv. The horizontal dotted line indicates a HI titer of 6 log_2_, which is the criterion for H9N2 LPAI vaccine efficacy in the Republic of Korea.

Serums were collected 3 wpv and 14 dpi. At 3 wpv, the log_2_ of the mean and standard deviation of HI tiers in the two vaccinated groups in the presence of homologous virus (LBM564) were 9.1 and 1.0, 9.8 and 1.0, respectively (Figure 2A). None of the sham groups had detectable HI titers before challenge. By 14 dpi, the log_2_ of the mean and standard deviation of HI tiers in the two vaccination groups were 9.5 and 1.0, 9.8 and 0.8, respectively. In antibody persistence, vac564 showed HI titers of >6 log_2_ (the criterion for H9N2 LPAI vaccine efficacy in Korea) until 24 wpv, although the mean antibody titers peaked at 3 wpv and decreased slightly (Figure 3).

#### 3.1.3. Virus Shedding and Replication Post-Challenge

To evaluate the efficacy of vac564, swab samples (OP and CL) and major tissues (lung, trachea, cecal tonsil, spleen, kidney, and liver) were collected and tested. When challenged with homologous virus (LBM564), the sham group shed virus on 1–7 dpi (10^1.4^–10^3.5^EID_50_/0.1 mL), whereas no virus shedding was observed in the vaccinated group (Table 3). However, vac564 did not prevent virus shedding against heterologous virus. When challenged with LBM314, the vaccinated group showed virus shedding on 1–5 dpi (10^2.4^–10^4.6^EID_50_/0.1 mL), although the virus shedding was significantly reduced on 3 dpi in CL swabs (*p* < 0.05). The virus shedding was detected in the sham group on 1–5 dpi (10^2.4^–10^5.2^EID_50_/0.1 mL).

Among the examined tissues, viral replication was detected in spleen and cecal tonsil from the sham group (10^1.4^–10^2.6^EID_50_/0.1 mL) when challenged with LBM564, but no viral replication was detected in any tissue from the vaccinated group (Table 4). In particular, the vaccine completely inhibited (100%) virus replication in cecal tonsil (*p* < 0.05), demonstrating vaccine efficacy against homologous lineage virus. By contrast, in LBM314 challenge, virus was detected in trachea, lung, and cecal tonsil at 10^1.0^–10^2.5^EID_50_/0.1 mL in the vaccinated group, while virus was detected in trachea, lung, and cecal tonsil (10^1.4^–10^2.2^EID_50_/0.1 mL) in the sham group. Moreover, the inhibition rate of the virus recovery was examined at 66.7% in cecal tonsil (i.e., 100%-positive detections rate of vaccinated group/positive detections rate of sham group), demonstrating that the vaccine did not protect against heterologous virus.

### 3.2. Study 2: Protection of Commercial G1 Lineage Vaccine against Challenge by Heterologous Viruses in SPF Chickens

#### 3.2.1. Serological Responses

Five-week-old SPF chickens were vaccinated with commercial G1 vaccine. At 3 wpv, the log_2_ of the mean and standard deviation of HI tiers in birds receiving the vaccine differed according to the heterologous viruses (LBM564 and LBM314; 1.3 ± 0.8 and 8.5 ± 0.7, respectively) (Figure 2B). None of the sham groups had detectable HI titers before challenge. By 14 dpi, the log_2_ of the mean and standard deviation of HI tiers in the two vaccination groups were 5.0 and 1.4, 10.3 and 1.1, respectively. SN assays were additionally conducted. As a result, SN titers of 32.5 ± 6.6 10 log_2_ and 92.5 ± 6.6 10 log_2_ were detected in the vaccinated groups at 3 wpv, with titers of 58.7 ± 10.5 10 log_2_ and 101.2 ± 5.9 10 log_2_ at 14 dpi (Figure 2C).

#### 3.2.2. Virus Shedding and Replication Post-Challenge 

To assess the ability of commercial G1 lineage vaccine against heterologous viruses, swab samples (OP and CL) and major tissues (lung, trachea, cecal tonsil, spleen, kidney, and liver) were used. In our study, vaccine did not prevent the virus shedding from H9N2 viruses of two heterologous viruses (Table 5). In LBM564 challenge, the sham group shed virus on 1–7 dpi (10^2.8^–10^4.5^EID_50_/0.1 mL), and virus was detected in the vaccinated group until 5 dpi, mostly in the OP samples (10^1.5^–10^3.5^EID_50_/0.1 mL), although the virus shedding was significantly reduced on 3–5 dpi in CL swabs (*p* < 0.01). In LBM314 challenge, virus titers in the sham group were 10^1.7^–10^4.6^EID_50_/0.1 mL on 1–7 dpi, while titers in the vaccinated group were 10^1.2^–10^4.8^ EID_50_/0.1 mL on 1–5 dpi; however, the virus shedding was significantly prevented in OP and CL swab samples of 1 and 3 dpi, respectively (*p* < 0.01). 

When challenged with LBM564, virus replication was not detected in all tested tissue samples in the vaccinated group, whereas virus replication was detected in the trachea, kidney, spleen, and cecal tonsil (10^0.6^–10^1.4^EID_50_/0.1 mL) in the sham group (Table 6). Similarly, when challenged with LBM314, no virus replication was observed in any tissue samples in the vaccinated group, while the sham group replicated virus in trachea, lung, kidney, and cecal tonsil (10^0.6^–10^2.6^EID_50_/0.1 mL).

## 4. Discussion

In this study, to prepare for the sudden emergence of other lineages of H9N2 viruses, we developed and evaluated a whole inactivated vaccine (vac564) via multiple passage of a recently identified Korean isolate. A commercial whole inactivated vaccine derived from the G1 lineage was imported and tested, as well.

In general, recombinant vaccine by reverse genetics which contain PR8 back-boned virus is known to be more easily replicated and proliferative in cell and egg culture systems [38,39]. Similarly, our previous studies demonstrated that rgH9N2/PR8 reassortant viruses show high productivity (10^8.3^ to 10^9.1^EID_50_/0.1 mL) [26,28]. Likewise, vac564, developed by multiple passage in eggs, also showed high productivity (10^8.4^EID_50_/0.1 mL and 1024 HA units at passage 30 (CE30)). This finding is consistent with a previous report showing that the conventional influenza H9N2 vaccine strain propagated stably with 10^8.7^EID_50_/0.1 mL at passage 20 [5]. These results indicate that the H9N2 vaccine generated by multiple passage in eggs has potential for use as a high-yield vaccine strain.

Generally, when used properly, potent avian influenza vaccines can prevent disease and death, increase resistance to infection, reduce replication and shedding of field viruses, and reduce virus transmission; however, they do not provide sterilizing immunity in the field [40]. Although various factors in the field can cause more severe disease than in the laboratory, resulting in differences in vaccine efficacy [2], vac564 prevented virus shedding in both OP and CL as well as 100% inhibition of virus replication in cecal tonsil after homologous challenge. This result is also consistent with those of a previous study showing that inactivated H9N2 vaccines provide high levels of protection against challenge with homologous virus, along with complete inhibition of virus shedding [41]. Thus, the findings presented herein indicate that vac564 developed by multiple passage in eggs is a potentially highly immunogenic and protective vaccine. 

Among the three lineages of H9N2 viruses, the G1 lineage is the most prevalent worldwide; however, it has never been isolated in Korea. In our study, we tested an imported G1 lineage commercial vaccine against heterologous viruses, due to absence of the parent virus. Antibody titers in response to both heterologous viruses varied, with a higher titer in response to the Y280 lineage virus than the Y439 lineage virus (HI titers of 8.5 ± 0.7 log_2_ and 1.3 ± 0.8, respectively); these results were confirmed by the HI and SN assays. This result is in agreement with that of a previous report showing that the nucleotide sequence homology between G1 lineage viruses and the Y439 lineage is 79.8–81.7% and between G1 lineage viruses and the Y280 lineage viruses is 86.6–90.45% [42]. Despite these antibody responses, there was no difference in protective efficacy in terms of virus shedding and replication. This result may be because the vaccine does not reduce virus shedding when the homology between the vaccine and the challenge virus is less than 90% [43]. When challenged with Y280 lineage virus, the difference between virus shedding in OP swab and replication in tissues indicates that the virus initially infects the upper respiratory tract but is insufficient to be replicated from lower respiratory tissues. Furthermore, challenge with homologous virus is required to determine the comprehensive efficacy of a G1 vaccine.

In summary, vac564 stimulated immune responses and provided SPF chickens with protection against challenge with a Y439 lineage virus but not a heterologous virus. The G1 lineage commercial vaccine inhibited replication of Y439 and Y280 lineage viruses but could not prevent shedding of either heterologous viruses. These findings suggest that considering genetic homology and appropriate vaccination is necessary, including those of the G1, Y280, and Y439 lineage viruses, through continuous genetic monitoring. Taken together, vaccine with a quick introduction should be prepared for a rapid control of H9N2 viruses.

## 5. Conclusions

Implementation of the H9N2 vaccination program in Korea has been effective at disease control, suggesting the need for application of suitable vaccines against newly emerging and re-emerging of G1 and Y439 lineage viruses in the future. In addition, genetic monitoring of viruses circulating in the field and timely updating of the vaccine seed strains should be considered to maintain control of outbreaks.

## Figures and Tables

**Table 1:** The multiple passage of LBM564 in 9–11-day-old embryonated specific-pathogen-free eggs.

Passage Number	LBM564	
	HA Unit	EID_50_/0.1 mL
CE2	16	10^6.5^
CE5	512	10^7.2^
CE10	512	10^8.2^
CE15	1024	10^7.7^
CE20	1024	10^7.5^
CE30	1024	10^8.4^
CE40	1024	10^8.5^

A vaccine strain based on LBM564 (Y439 lineage) was developed by multiple passage in 9–11-day-old embryonated specific-pathogen-free eggs. Abbreviations: HA, hemagglutination activity; EID_50_, 50% egg infectious dose.

**Table 2:** Hemagglutination inhibition titers generated by vac564 in specific-pathogen-free (SPF) chickens.

Vaccine	Vaccination			HI Titer (log_2_)	
	No. of Chickens	Vaccine Dose (EID_50_/0.1 mL)	Vaccine Route	2wpv	3wpv
vac564	8	10^8.0^	IM	7.0 ± 1.2 *	8.0 ± 1.2 *
		10^7.5^		1.4 ± 1.3 **	2.9 ± 2.1 **
Sham		PBS		- ^1^	-

Serum was collected at 3 weeks post-vaccination (wpv), and the titers of antibody against homologous virus were determined in hemagglutination inhibition (HI) assays using 1% chicken RBCs. The minimum immunizing dose was 10^8.0^EID_50_/0.1 mL, which showed a titer of 8.0 ± 1.2 log_2_ at 3 wpv in SPF chickens. The values were analyzed with Student *t*-test. * *p* > 0.05, ** *p* > 0.05. ^1^ The dashes (-) indicate no measurable titer (<3 log_2_ of HI titer). Abbreviations: No, number; EID_50_, 50% egg infectious dose; IM, intramuscularly; PBS, phosphate buffer saline.

**Table 3:** Virus shedding by vaccinated SPF chickens challenged with Y439 and Y280 lineage H9N2 viruses (vac564).

Group	Vaccine	No. of Chickens ^1^ (HI Titer at 3 wpv, log_2_)	Challenge Virus (Lineage)	Virus Shedding (log_10_EID_50_/0.1 mL)											
				1dpi		3dpi		5dpi		7dpi		10dpi		14dpi	
				OP	CL	OP	CL	OP	CL	OP	CL	OP	CL	OP	CL
Vaccinated	vac564	8/8 (9.1 ± 1.0)	LBM564 (Y439)	0/8 ^3,^** (-) ^4^	0/8 (-)	0/8 ** (-)	0/8 (-)	0/8 ** (-)	0/8 (-)	0/8 (-)	0/8 (-)	0/8 (-)	0/8 (-)	0/8 (-)	0/8 (-)
Sham		0/8 (-) ^2^		7/8 (2.8)	0/8 (-)	8/8 (3.5)	1/8 (1.4)	8/8 (2.9)	3/8 (3.5)	0/8 (-)	2/8 (3.2)	0/8 (-)	0/8 (-)	0/8 (-)	0/8 (-)
Vaccinated		8/8 (9.8 ± 1.0)	LBM314 (Y280)	8/8 (3.6)	0/8 (-)	8/8 (4.6)	0/8 * (-)	8/8 (2.4)	0/8 (-)	0/8 (-)	0/8 (-)	0/8 (-)	0/8 (-)	0/8 (-)	0/8 (-)
Sham		0/8 (-)		7/8 (3.8)	0/8 (-)	8/8 (5.2)	4/8 (2.4)	8/8 (3.2)	0/8 (-)	0/8 (-)	0/8 (-)	0/8 (-)	0/8 (-)	0/8 (-)	0/8 (-)

The values were analyzed with Fisher’s exact test. * *p* < 0.05, ** *p* < 0.01. Abbreviations: HI, hemagglutination inhibition; EID_50_, 50% egg infectious dose; wpv, weeks post-vaccination; OP, oropharyngeal; CL, cloacal. ^1^ Number of antibody-positive chickens/Total number of chickens. ^2^ Dashes indicate no measurable titer (<3 log_2_ of HI titer). ^3^ Number of positive samples/Total tested. ^4^ Dashes indicate no measurable titer (<10^1.0^ EID_50_/0.1 mL).

**Table 4:** Virus replication in tissues from vaccinated SPF chickens challenged with Y439 and Y280 lineage H9N2 viruses (vac564).

Group	Vaccine	Challenge Virus (Lineage)	Virus Replication in Tissues (log_10_EID_50_/0.1 mL)					
			Trachea	Lung	Kidney	Spleen	Liver	Cecal Tonsil
Vaccinated	vac564	LBM564 (Y439)	0/8 ^1^ (-) ^2^	0/8 (-)	0/8 (-)	0/8 (-)	0/8 (-)	0/8 * (-)
Sham			0/8 (-)	0/8 (-)	0/8 (-)	1/8 (1.4)	0/8 (-)	4/8 (2.6)
Vaccinated		LBM314 (Y280)	4/8 (2.5)	1/8 (1.2)	0/8 (-)	0/8 (-)	0/8 (-)	1/8 (1.0)
Sham			6/8 (2.2)	1/8 (1.4)	0/8 (-)	0/8 (-)	0/8 (-)	3/8 (1.5)

The values were analyzed with Fisher’s exact test. * *p* < 0.05. Abbreviations: EID_50_, 50% egg infectious dose. ^1^ Number of positive samples/Total tested. ^2^ Dashes indicate no measurable titer (<10^1.0^ EID_50_/0.1 mL).

**Table 5:** Virus shedding by vaccinated SPF chickens challenged with Y439 and Y280 lineage H9N2 viruses (G1).

Group	Vaccine	No. of Chickens ^1^ (HI Titer at 3 wpv, log_2_)	Challenge Virus (Lineage)	Virus Shedding (log_10_EID_50_/0.1 mL)											
				1dpi		3dpi		5dpi		7dpi		10dpi		14dpi	
				OP	CL	OP	CL	OP	CL	OP	CL	OP	CL	OP	CL
Vaccinated	G1	8/8 (1.3 ± 0.8)	LBM564 (Y439)	8/8 ^3^ (2.6)	0/8 (-) ^4^	8/8 (2.2)	0/8 ** (-)	6/8 (3.5)	1/8 ** (1.5)	0/8 (-)	0/8 (-)	0/8 (-)	0/8 (-)	0/8 (-)	0/8 (-)
Sham		0/8 (-) ^2^		8/8 (2.8)	0/8 (-)	8/8 (2.8)	7/8 (4.1)	7/8 (3.5)	7/8 (4.5)	0/8 (-)	2/8 (2.8)	0/8 (-)	0/8 (-)	0/8 (-)	0/8 (-)
Vaccinated		8/8 (8.5 ± 0.7)	LBM314 (Y280)	0/8 ** (-)	0/8 (-)	3/8 (4.8)	0/8 ** (-)	7/8 (1.5)	1/8 (1.2)	0/8 (-)	0/8 (-)	0/8 (-)	0/8 (-)	0/8 (-)	0/8 (-)
Sham		0/8 (-)		7/8 (3.8)	0/8 (-)	6/8 (4.6)	7/8 (2.6)	8/8 (3.6)	4/8 (2.4)	0/8 (-)	1/8 (1.7)	0/8 (-)	0/8 (-)	0/8 (-)	0/8 (-)

In the G1 vaccination experiment, only heterologous viruses were used for challenge because no homologous virus was isolated in Korea. The values were analyzed with Fisher’s exact test. ** *p* < 0.01. Abbreviations: HI, hemagglutination inhibition; EID_50_, 50% egg infectious dose; wpv, weeks post-vaccination; OP, oropharyngeal; CL, cloacal. ^1^ Number of antibody-positive chickens/Total number of chickens. ^2^ Dashes indicate no measurable titer (<3 log_2_ of HI titer). ^3^ Number of positive samples/Total tested. ^4^ Dashes indicate no measurable titer (<10^1.0^ EID_50_/0.1 mL).

**Table 6:** Virus replication in tissues from vaccinated SPF chickens challenged with Y439 and Y280 lineage H9N2 viruses (G1).

Group	Vaccine	Challenge Virus (Lineage)	Virus Replication in Tissues (log_10_EID_50_/0.1 mL)					
			Trachea	Lung	Kidney	Spleen	Liver	Cecal Tonsil
Vaccinated	G1	LBM564 (Y439)	0/8 ^1^ (-) ^2^	0/8 (-)	0/8 (-)	0/8 (-)	0/8 (-)	0/8 (-)
Sham			1/8 (1.4)	0/8 (-)	1/8 (-)	1/8 (1.0)	0/8 (-)	2/8 (1.2)
Vaccinated		LBM314 (Y280)	0/8 ** (-)	0/8 ** (-)	0/8 * (-)	0/8 (-)	0/8 (-)	0/8 (-)
Sham			7/8 (2.6)	6/8 (1.2)	5/8 (-)	0/8 (-)	0/8 (-)	1/8 (1.0)

In the G1 vaccination experiment, only heterologous viruses were used for challenge because no homologous virus was isolated in Korea. The values were analyzed with Fisher’s exact test. * *p* < 0.05, ** *p* < 0.01. Abbreviations: EID_50_, 50% egg infectious dose. ^1^ Number of positive samples/Total tested. ^2^ Dashes indicate no measurable titer (<10^1.0^ EID_50_/0.1 mL).

## Data Availability

The data generated during this study are available in this published article.
